# Machine Vision System for Automatic Adjustment of Optical Components in LED Modules for Automotive Lighting

**DOI:** 10.3390/s23218988

**Published:** 2023-11-05

**Authors:** Silvia Satorres Martínez, Diego Manuel Martínez Gila, Sergio Illana Rico, Daniel Teba Camacho

**Affiliations:** 1 Robotics, Automation and Computer Vision Group, Electronic and Automation Engineering Department, University of Jaén, 23071 Jaén, Spain; satorres@ujaen.es (S.S.M.); sillana@ujaen.es (S.I.R.); 2 Valeo Lighting Systems, 23600 Jaén, Spain; daniel.teba@valeo.com

**Keywords:** machine vision, automatic adjustment, LED module, headlamp, automotive lighting

## Abstract

This paper presents a machine vision system that performs the automatic positioning of optical components in LED modules of automotive headlamps. The automatic adjustment of the module is a process of great interest at the industrial level, as it allows us to reduce reworks, increasing the company profits. We propose a machine vision system with a flexible hardware–software structure that allows it to adapt to a wide range of LED modules. Its hardware is composed of image-capturing devices, which enable us to obtain the LED module light pattern, and mechanisms for manipulating and holding the module to be adjusted. Its software design follows a component-based approach which allows us to increase the reusage of the code, decreasing the time required for configuring any type of LED module. To assess the efficiency and robustness of the industrial system, a series of tests, using three commercial models of LED modules, have been performed. In all cases, the automatically adjusted LED modules followed the ECE R112 regulation for automotive lighting.

## 1. Introduction

LED-based lighting systems have been extensively used in the automotive industry for more than 30 years. Initially, they were used for interior lighting and rear signalling functions, but the continuous progress of optoelectronic technology made them a viable alternative for forward lighting [1]. Due to their reliability, low power consumption, compact size, and long life time, white light LEDs (WLLED) are becoming a popular source for headlamp illumination [2,3].

One of the main barriers to use WLLED sources in vehicle forward lighting was the luminous efficiency, but in 2006, it was exceeded the limit of 100 lm/W, enabling their use in this application [4]. For the first time in 2007, a luxury car was equipped with an LED headlamp [5]. This was the beginning of a new era in vehicle front lighting, and nowadays, this technology is outpacing other options such as Xenon or incandescent bulbs [6].

The main function of a headlamp is to illuminate all objects on the roadway, avoiding inducing glare to drivers or pedestrians. To this end, headlamps project a light pattern that must follow the Economic Commission for Europe (ECE) R112 regulation [7]. Light patterns have to meet the luminous intensities defined in the ECE R112 at certain test points. In a headlamp, light patterns are generated by the LED module [8]. One of its optical components is the thick lens, which is made of plastic [9]. Due to the high volume of demand for modules, thick lenses are manufactured by injection molding and specifically in multi-cavity molds. Even if identical parts are injected at the same time, the tolerances in the different cavities cause dimensional deviations among them [10,11]. This has a significant bearing on the photometric results of the module.

As a result of the above, the mass production of modules require high precision manufacturing and assembling of the optical components included in them. Any dimensional variation in its optical components has an influence on the final module assembly. LED modules are currently assembled to a fixed position. This implies that when there are dimensional variations in their optical components, most of them have to be rejected and manually adjusted to meet with the ECE regulation.

The above confirms the industry’s need for an automatic adjustment system capable of optimally assembling LED modules. The system should also comply with the following requirements:

- Reliability and robustness: The hardware devices and the software algorithms used to obtain the optimal fixing position and adjust the module should guarantee that it complies with the regulation.
- Capability for real-time adjustment: Since the automatic adjustment system is one element of the production chain, the process must be performed in the pre-established amount of time. These cycle time requirements will significantly affect the positioning algorithms.
- Adaptability: The automated adjustment system should be easily adapted to different models of LED modules. For this reason, it is absolutely necessary that the system’s components may be included, eliminated, or even modified with ease.

With the intention of achieving this goal, we introduce a novel automatic adjustment system designed for LED modules. This system possesses versatility and can be tailored to various part models. The system follows a component-based development approach [12], which allows us to combine commercially available components (programmable logic controller (PLC) control software and computer vision algorithms) with custom-made ones (very specific image processing algorithms, data presentation strategies, etc.). From an academic point of view, this MVS design approach is valuable for new devices to be integrated into an Industry 4.0 environment and its new paradigm, Zero Defect Manufacturing [13,14].

The remainder of this paper is as follows. First, the definition of the problem and related works are introduced in Section 2. Section 3 details the design of the machine vision system, analysing its hardware and software architectures. The experimental validation of the system, using a commercial LED module, is presented in Section 4. The paper ends with the conclusions given in Section 5.

## 2. Definition of the Problem and Related Work

In this section, we describe the structure of an LED module and how it is currently assembled to meet the criteria defined in the regulation. We also list previous proposals that address a similar issue: the automatic assembly of parts with dimensional variations.

### 2.1. LED Modules for Automotive Lighting

LED-based automotive headlamps include a variety of lighting functions such as low beam, high beam, daytime running light, and fog beam. Basically, these lighting functions follow two types of lighting patterns and what mainly differentiates them is the inclusion or non-inclusion of the cut-off line [6]. The principle of the cut-off line is to protect people by decreasing glare on the roadway. The low beam and the fog beam require a high-contrast cut-off line, while the high beam and the daytime running light are without it.

**Figure 1:**
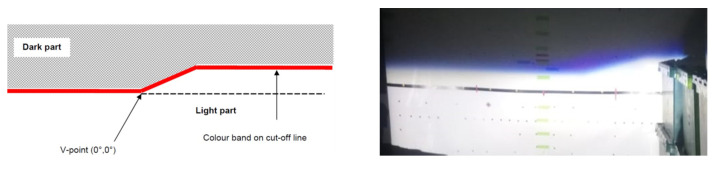
The low-beam lighting pattern. Left: the cut-off line shape; right: low-beam projection in an inspection tunnel.

Regarding illumination patterns, the high beam provides a clear vision to the driver but should only be used to illuminate far-off distances where visibility is mostly low. Its lighting pattern is an ellipse and, because of this, it induces serious glare to people on the roadway. The low beam pattern is much more complicated because of the cut-off line (Figure 1). As can be seen, the dark part above the cut-off line should provide limited illumination to avoid glare. The bright part, below the cut-off line, should cover the important viewing zone for the driver.

**Figure 2:**
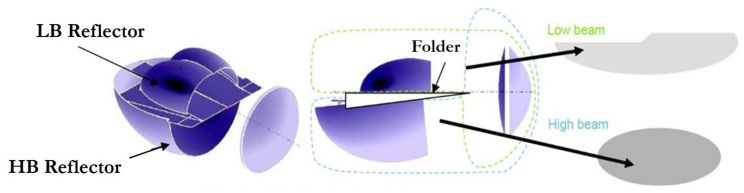
LED module of a headlamp producing both low- and high-beam light “BiLED module”.

Currently, there are automotive headlamp models that combine the low beam and high beam in a single LED module [15,16]. Some automotive lighting companies refer to this concept as BiLED [17,18] and it is mainly characterised by its compactness and reduced mechanical complexity. Figure 2 shows the BiLED concept developed in 2015 by one of these companies [17]. In this design, the light source comprises high power multichip LEDs arranged on a two-layered PCB and three optical active components responsible for the light beam distribution. These optical components are (i) the reflector collector, to recover and address the LED light flow; (ii) the folder mirror, to define the focal plane; and (iii) the lens, to deploy the light distribution on the road. A plastic frame assures the relative position of these three optical components and the PCB.

**Figure 3:**
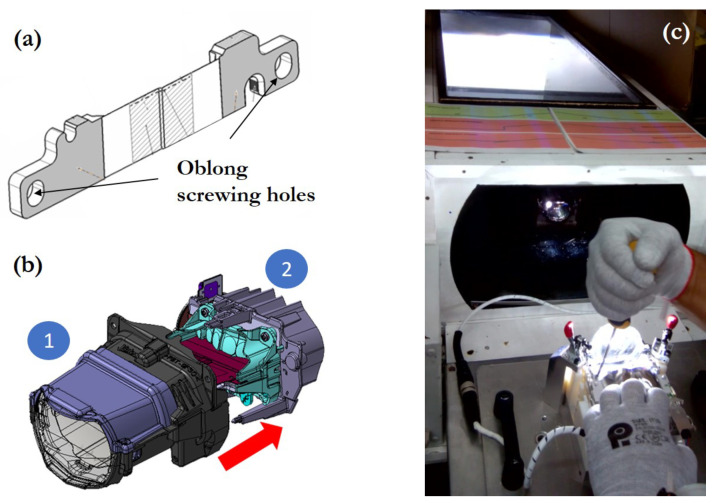
LED module and manual positioning: (**a**) folder; (**b**) submodules in the elliptical module: (1) lens, lens support, and housing; (2) heatsink, flex board, collimators, and folder; and (**c**) LED module rework station.

It must be noted that the dimensional variability of each individual component can affect the optimal location of the focal plane. This variability can be corrected with a specific assembly position for each module. This is why the folder was designed having oblong screwing holes, allowing it to find an optimal relative position between submodules (Figure 3a,b).

#### Manual Positioning and Quality Control

In the mass production of BiLED modules, nonconformities are detected due to the manufacturing process of the active optical components and the processes associated with their assembly. In addition to not complying with the current regulations, the color of the cut-off line may be the reason for the rejection of the part. Cut-off colors that are too bluish or reddish make the modules unacceptable to the customer. The rejected BiLED modules can be reworked in special working stations as the one shown in Figure 3c. These stations where designed and constructed for a manual positioning of the folder according to a visual inspection of the cut-off projection. This manual positioning process had the following steps:

- Remove the parts that prevent access to the active optical components.
- Loosen the screws securing the optical component.
- Switch on the module and project the light beam onto a front screen.
- Manually adjust the relative position of the optical components according to the aspect of the projected image. This step was subjected to the limitations of visual perception and the fatigue caused by the observation of light distributions with strong light–dark contrast.
- Once the correct position of the components has been found (limited by the poor visual precision of the human eye), lock this position by screwing on the optical part.
- Mount and screw the rest of the non-functional components of the module.
- Check the photometry of the module on the photometric bench again.

If after the manual readjustment steps the result was compliant, the module was labelled and packaged for assembly inside the headlamp; however, if the result was not compliant, all the readjustment steps had to be performed again. All of this led to an increase in time and cost overruns in the production of the module.

The above mentioned justifies the need for an automatic positioning system capable of obtaining the optimum relative position of the module’s optical components and their attachment to each other. The automatic adjustment system will eliminate module reworks, increasing the efficiency of the production process of BiLED modules.

### 2.2. Related Works on Automatic Assembling of Parts with Dimensional Variations

Assembly is one of the most important stages of a company’s entire manufacturing process. It is especially critical in certain industries such as aeronautics or automotive, where products with high precision requirements are manufactured [19]. The final assembly accuracy of products is dependent on many factors such as the dimension and geometric tolerances of the parts to be assembled, the assembly clearance of parts, the load deformation, the deformation caused by residual stress release, and the adjustment of the assembly process [20].

As the assembly process directly affects the product quality, it is very important to carry out an assembly precision analysis and optimisation. In this regard, there are numerous contributions that propose models for achieving assembly precision requirements at a low manufacturing cost. In [21], a semantic-based assembly precision optimisation method was proposed considering the process capacity illustrated via an aircraft inner flap. The trade-offs between the cost and achievable variation limits of the entire manufacturing chain was studied in [22]. A method based on a general part digital twin model was presented in [23] to improve the reliability of the assembly precision. The proposal considered other factors that are usually ignored, such as the assembly-positioning error and the deformation of the mating surface.

One way to increase the assembly precision of a product is to reduce the tolerance range of the parts. However, this cannot be reduced unlimitedly and it can lead to higher costs [24]. A clear example of this problem are the plastic parts, which are increasingly being used as the main elements of diverse commercial products. One of the most widespread drawbacks of these types of parts is their dimensional variations or manufacturing tolerances, especially in ones with complex geometry or considerable size [10]. This issue was analysed in [25], where a prototype for assembling the cover lens and the housing of vehicle headlamps was presented. After analysing different technologies, a fuzzy controller was implemented, which, based on the data provided by laser and micrometric contact sensors, gave orders to linear magnetic actuators to fix both parts.

According to the previous works reviewed, the assembly precision optimisation process contains a wealth of expert experience and knowledge which can be used as important information sources. Our proposal for an automatic adjustment system for LED modules is based on the manual position system which is utilised for reworks. As with the manual system, measurements and adjustments are required for the assembly process to ensure that the LED module follows the ECE R112 regulation and the specific requirements defined by the customer.

## 3. Description of the Machine Vision System

**Figure 4:**
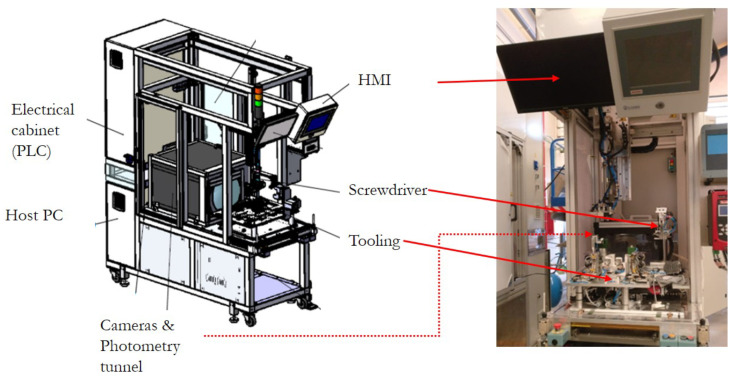
Machine vision system for the automatic precision assembly of LED modules.

A Machine Vision System (MVS) for the precision assembly of LED modules (Figure 4) has been designed, constructed, and programmed. The automatic assembly is based on a measurement and adjustment procedure. Measurements are obtained from the hardware devices to acquire the low-beam lighting pattern projection at 25 m (photometry devices) and the image processing algorithms to obtain the required information for the control algorithm. The assembly optimal position is obtained via the control algorithm based on the reference, which is previously defined via a calibration procedure. In this section, we present the MVS hardware and software architectures. Some diagrams of the Unified Modelling Language (UML), a widely adopted tool for system engineering modelling [26], implement the MVS description.

### 3.1. Hardware Architecture

**Figure 5:**
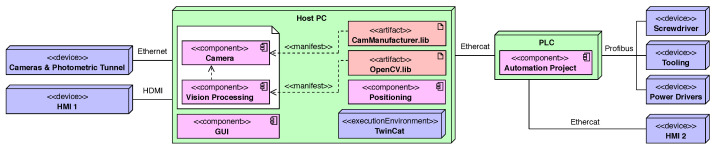
Deployment diagram describing the hardware architecture.

The hardware architecture shown in Figure 5 uses a UML deployment diagram. This diagram shows the MVS hardware, the software running on this hardware, and the interconnected networks that facilitate communication between various nodes. Basically, the MVS consists of the following hardware elements:

- Camera and photometric tunnel.
- A Host PC to synchronise all the processes, execute the computer vision, and position the control algorithms.
- Tooling for holding the modules and allowing the relative movement between submodules.
- Screwdriver to fix the submodules.
- A PLC to control the movement of elements and the lighting of the LED module.
- Power drivers of the module.
- HMI screens to show results and tune parameters.

The camera and the photometric tunnel are hardware devices which interfaces with the *Camera* software component. This software component is in charge of acquiring and processing the image. For the image acquisition, the libraries provided by the camera manufacturer are required. For the processing, specific libraries such as the OpenCV and proprietary computer vision algorithms are needed.

The MVS includes a series of elements which allow us to position and assemble the LED submodules. These elements are the tooling and the screwdriver. Both interfaces with the software component *Positioning* and are controlled by a PLC connected via EtherCAT to the host PC using the TwinCAT environment. The PLC also controls the lighting of the LED module through the power drivers.

Two HMI screens are part of the MVS hardware architecture. The $HMI_{1}$ screen interfaces with the component software *GUI*. This component enables us to show results and tune the camera and processing parameters. The $HMI_{2}$ screen interfaces with the PLC component *AutomationProject*. Through this component, it is possible to directly control and move the devices that manage the relative movement and adjustment of submodules. In addition, the finite state machine is implemented which controls the order of execution of the software components and how they relate. We next present a further description of the image acquisition and position adjustment devices.

#### 3.1.1. Image Acquisition Devices

**Figure 6:**
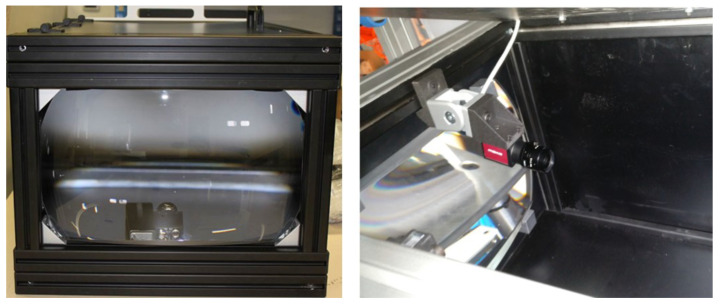
Image acquisition devices. The photometric tunnel and the colour camera inside the tunnel.

The devices related to the image acquisition are the vision sensor and the photometric tunnel (Figure 6). The vision sensor is one Ethernet Basler colour camera with a 1.3 Mp resolution and an acquisition rate of 30 fps. It is used for the study of the regions of interest of the low-beam pattern. The photometric tunnel allows us to acquire the lighting pattern projection at 25 m. It includes a lens, a background panel, and the necessary tool for the vision sensor placement. In addition, it is a closed structure that prevents any type of light contamination during the image acquisition.

#### 3.1.2. Position Adjustment Devices

**Figure 7:**
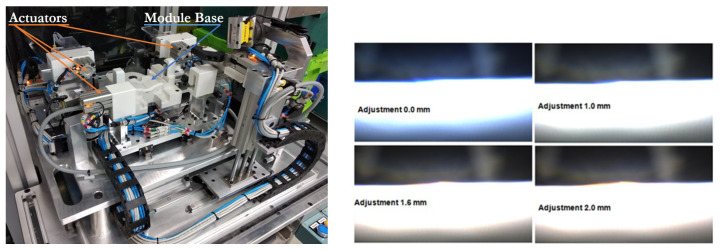
Tooling and the cut-off light pattern for a sample of the eleven discrete positions of the folder.

The tooling is the device where the relative movement between submodules takes place (Figure 7). Since the precision for the folder movement is two-tenths of a millimetre and the oblong screwing holes allow a displacement of 2 mm, eleven discrete positions are available for the assembling. With each of these eleven positions, a different pattern of cut-off light is obtained. Once the best position is achieved, the folder screws are fixed using a three-axis screwdriver.

### 3.2. Software Architecture

**Figure 8:**
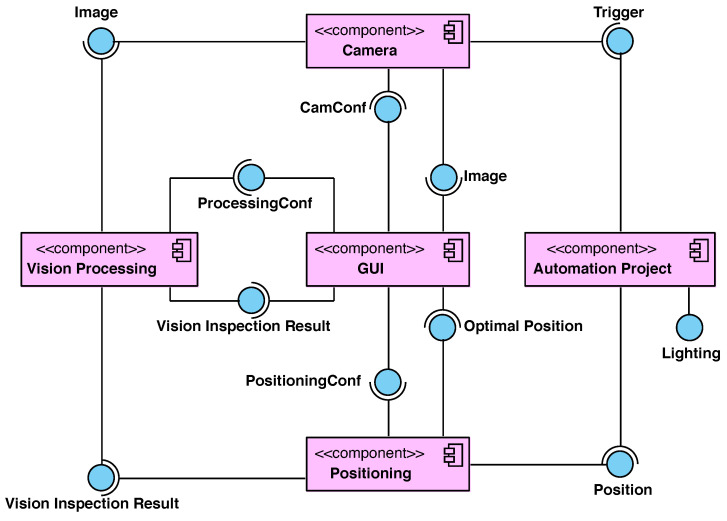
Component diagram describing the software architecture.

The MVS software architecture follows a component-based development approach [27] which is illustrated in the UML component diagram presented in Figure 8. This architecture enables the MVS to be versatile, open, and easy to maintain.

The component which provides and requires a greater number of interfaces is the *GUI*. This component has six interfaces: *Image*, *Optimal Position*, *Vision Inspection Result*, *CamConf*, *ProcessingConf*, and *PositioningConf*. The first three interfaces are required services while the last three ones are offered services. Through the required services, the component receives the image of the cut-off light pattern (*Image*), provided by the *Camera*; its processing (*Vision Inspection Result*), computed by the *Vision Processing*; and the optimal position of the folder, obtained by the *Positioning*. The *GUI* offers configuration parameters to the aforementioned three components.

The *Automation Project* controls the order of execution. Through the offered services, *Trigger* and *Lighting*, the component switches the BiLED module and triggers the camera to start the image acquisition. The required service is *Position*, provided by the *Positioning*. Each time it receives a new position, the relative position of the BiLED submodules is modified by the tooling. When the position does not vary between two consecutive iterations, it means that the optimal position has been reached and the assembling concludes.

The *Positioning* requires the services, *Vision Inspection Result* and *PositioningConf*, to offer *Position* and *Optimal Position*. The *Vision Processing* requires *Image* and *ProcessingConf* services to offer *Vision Inspection Result*. The main algorithms, executed in both components, are detailed below.

#### 3.2.1. Image Processing

The computer vision algorithms have to perform photometric and chromatism measurements of the low-beam pattern. Basically, these measurements are the cut-off sharpness and the cut-off color. In order to match the ECE R112 regulation, the low-beam pattern is required to contain a clear cut-off line, which has to achieve a certain standard in illumination contrast. In addition, the customer imposes limits to the cut-off colour which must be met for the LED module to be accepted. How to obtain both measurements is detailed below, as well as the previously needed calibrations.

**Figure 9:**
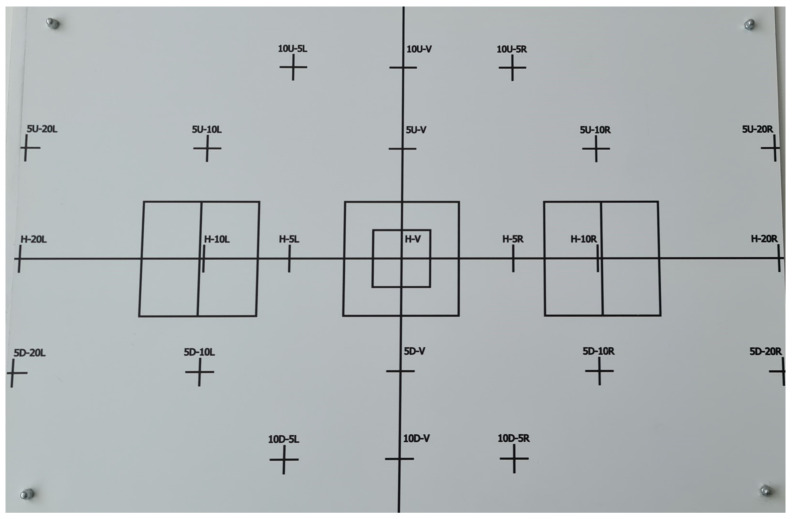
Calibration pattern used to perform the geometric calibration by the MVS.

**Figure 10:**
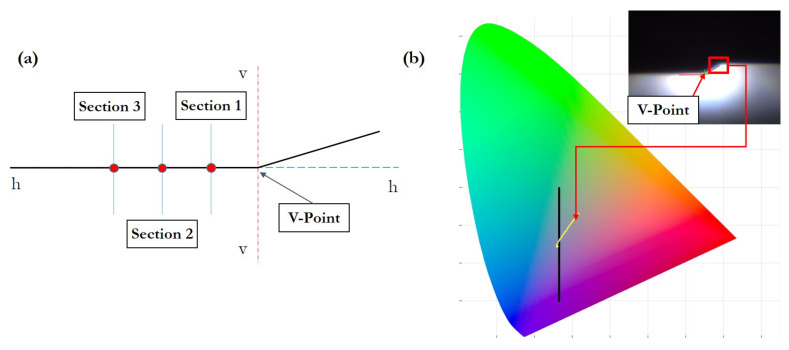
Measurement obtained from the cut-off pattern: (**a**) measured sections on the left side of the V point used to obtain the cut-off sharpness; (**b**) color measurements in the CIE diagram from points placed on the border of the cut-off line.

- Calibrations.
  - Photometric. This calibration establishes a correlation between the gray level of the image and a standardised illuminance value. A procedure is established using a standard module to apply corrections to the intensity values acquired by the vision sensor.
  - Geometric. This calibration determines the relationship between the pixels in an image and sexagesimal degrees in spherical coordinates. To achieve this, a calibration pattern (Figure 9) is placed on the background panel of the photometric tunnel. The calibration pattern is placed at a distance of 700 mm and the length of the square in the center measures 43 mm. The pattern is acquired by the vision sensor and processed to obtain the length of the square in pixels (170 pixels). The desired pixel–degree ratio can be obtained from:
    (1) $$n_{p}=\frac{700\cdot 170}{43}\cdot \tan\theta ,$$
    where $n_{p}$ is the number of pixels between two points in the image, and θ is the angle formed by these two points in spherical coordinates.
- Cut-off measurements. For these measurements, the V point in the horizontal part of the cut-off line has to be located (Figure 10b). Traditional image processing algorithms are used for this. As the gray level image shows a bimodal histogram with a homogeneous background, the Otsu algorithm is used for segmentation [28]. Then, via the Hough transform, the cut-off line shape is extracted [29]. The lower intersection point is the desired V Point.
  - Sharpness. It is determined by vertically scanning three sections placed through the horizontal part of the cut-off line (at H 1.5${}^{\circ }$, 2.5${}^{\circ }$, and 3.5${}^{\circ }$, Left or Right, depending on LHD/RHD from the V point). During the scanning, the gradient *G* is determined using the formula:
    (2) $$G=logE_{\beta }-logE_{\left(\beta +\delta \right)}$$
    where *E* is the illuminance, β is the vertical position in degrees, and δ is the scan resolution, also in degrees. The sharpness of the cut-off line is the point with the highest gradient with respect to the H-H axis.
  - Chromatism. The low beam color has to be inside the specific limits defined in the CIE diagram. Figure 10b shows the limit specified by the customer in the CIE diagram. RGB values of the pixels inside the ROI in the cut-off line are changed to the XYZ CIE coordinates. Only X and Y coordinates are taken into account to place the RBG values in the CIE diagram. The customer defines the black line in the CIE diagram and a specified percentage of points should remain on the left (% blue) and the others should remain on the right (% red).

#### 3.2.2. Optimal Position Adjustment

**Figure 11:**
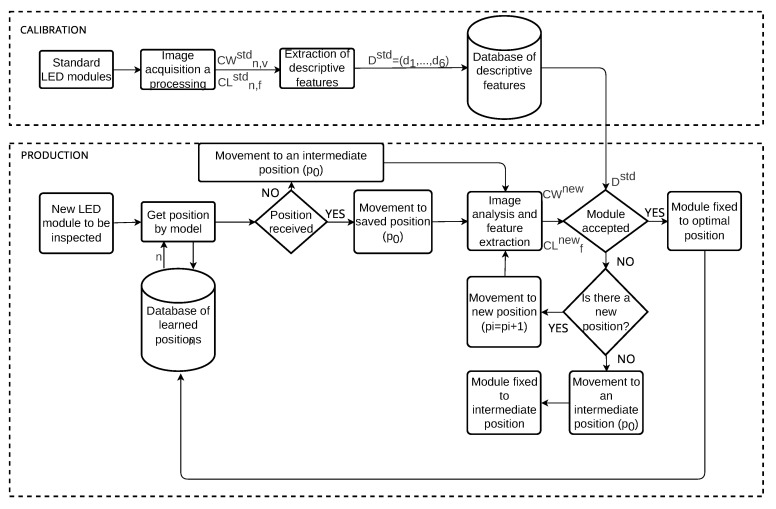
Algorithm for the optimal position adjustment.

Figure 11 shows a flow diagram of the designed algorithm to achieve the optimal position in which the LED module has to be assembled and adjusted. First, to determine this position, it is necessary to carry out a calibration phase. In this phase, already assembled modules, which have been validated in a photometric tunnel and meet with the regulation, are placed on the MVS tooling. Modules are lighted with the purpose of obtaining the cut-off measurements with the MVS. According to the previous description of the image processing algorithms, these measurements are the sharpness and the chromatism. The sharpness can be mathematically modelled as $CW_{n,v}^{std}$, where *n* is the number of modules used in the calibration phase and *v* indicates each of the three vertical lines over which the maximum gradient is obtained. The colour features for the chromatism are stored in the array $CL_{n,f}^{std}$, where *f* denotes each of the two features considered to measure the low beam colour.

Subsequently, six characteristics describe the LED modules used for calibration ($D^{std}$). The first two are related to the cut-off sharpness (Equations (3) and (4)) and the remaining four to the chromatism (Equations (5)–(8)):

(3) $$d_{1}^{std}=min\left({\bar{CW}}^{std}\right)$$

(4) $$d_{2}^{std}=max\left({\bar{CW}}^{std}\right)$$

(5) $$d_{3}^{std}=min\left({\bar{CL}}_{1}^{std}\right)$$

(6) $$d_{4}^{std}=max\left({\bar{CL}}_{1}^{std}\right)$$

(7) $$d_{5}^{std}=min\left({\bar{CL}}_{2}^{std}\right)$$

(8) $$d_{6}^{std}=max\left({\bar{CL}}_{2}^{std}\right)$$

where the variables ${\bar{CW}}^{std}$, ${\bar{CL}}_{1}^{std}$, and ${\bar{CL}}_{2}^{std}$ correspond to the equations (Equations (9)–(11)).

(9) $${\bar{CW}}^{std}=\frac{\sum_{n=1}^{N}\sum_{v=1}^{4}CW_{n,v}^{std}}{N\cdot 3}$$

(10) $${\bar{CL}}_{1}^{std}=\frac{\sum_{n=1}^{N}CL_{n,1}^{std}}{N}$$

(11) $${\bar{CL}}_{2}^{std}=\frac{\sum_{n=1}^{N}CL_{n,2}^{std}}{N}$$

Second, the optimal position adjustment procedure for new modules is carried out in the production phase. This position has to be reached according to the following sequence:

- Step 0: Check if the LED module model has a previously learned optimal position. To do this, a query is made on the database of learned positions. If the model already has a registered position, this is the initial one. If not, it is initialised to an intermediate position. The position vector is initialised as follows:
  (12) $$p_{i}=\left\{\begin{array}{c} p_{0}=n\\ p_{1}=p_{0}+1\\ p_{2}=p_{0}-1\\ p_{i}=p_{i-2}+1\;\mathrm{when}\;i>2\;\&\;i=\left(2k+1\right)\forall k\in \left(1,...\right)\\ p_{i}=p_{i-2}-1\;\mathrm{when}\;i>2\;\&\;i=\left(2k\right)\forall k\in \left(2,...\right) \end{array}\right.$$
  where *n* is the initial position (learned or intermediate).
- Step 1: Set the LED module to the position $p_{i}$. First time i=0. If $p_{i}$ is outside the position range allowed by the tooling, the module is adjusted in the intermediate tooling position. For this module, it is not possible to reach an optimal position, so go to Step 4. If it is within the range, go to Step 2.
- Step 2: Acquire and process the cut-off shape image to obtain the cut-off measurements: $CW^{new}$, $CL_{1}^{new}$, and $CL_{2}^{new}$.
- Step 3: If $CW^{new}\in \left(d_{1}^{std},d_{2}^{std}\right)$ and $CL_{1}^{new}\in \left(d_{3}^{std},d_{4}^{std}\right)$ and $CL_{2}^{new}\in \left(d_{5}^{std},d_{6}^{std}\right)$, the module has been correctly adjusted and assembled and the position is recorded in the database; if so, go to Step 4. If not $p_{i}=p_{i+1}$, then go to Step 1.
- Step 4: Completion stage. Result notification via HMI.

## 4. Experimental Case of Study: BiLED Module

The experimental validation was conducted with commercial BiLED modules. A total of 112 modules of three different models were used for the calibration phase. For the production phase, 134 modules were used, also belonging to the three models used for the calibration. Both phases are detailed below.

### 4.1. Calibration Phase

To carry out the calibration, as a starting point, modules adjusted to the customer’s requirements and validated in a photometric bench were taken as a reference. Specifically, the three models of BILED modules identified as TD-IZQ, IO1-TI-IZQ, and F56-USA-IZQ. A total number of 112 modules were used for this task (40, 48, and 24 modules for each model, respectively). The BiLED models and the number of them were selected by the customer based on his specific necessities. Table 1 shows the features cut-off sharpness, percentage of blue, and percentage of red obtained by the MVS for each of the former modules.

Based on the parameters extracted from each module, the acceptance ranges for each of the models were obtained. Table 2 shows these acceptance ranges. As can be seen, the most critical ranges of acceptance are those of the model F56-USA-IZQ. This means that, for this model, it will be more complicated for the MVS to reach an optimal position.

### 4.2. Production Phase

In this phase, BiLED modules were automatically adjusted and assembled by the MVS. The optimal assembly position and the cut-off measurements, for the reached positions, are shown for each of the BiLED models in Table A1, Table A2 and Table A3. For each of the tables, the columns present the following information: module ID, the cut-off sharpness, the chromatism analysis (% red and % blue), and the optimal position. If it is not possible to find an optimal assembly position for the module within the acceptance range, it is represented as “-”. In this case, and according to the positioning algorithm, the module is assembled in an intermediate position and it is separated for a subsequent manual rework or rejection.

The MVS performance was tested with 134 BiLED modules belonging to the following models: TD-IZQ model (58), IO1-TI-IZQ (37), and F56-USA-IZQ (39). A detailed description of the results of the production phase is now given and discussed.

**Figure 12:**
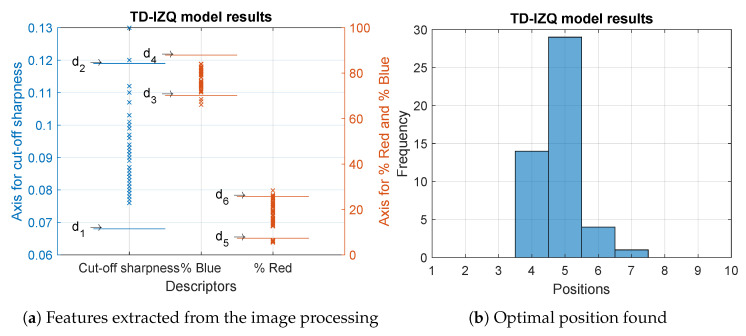
Results of the analysed modules belonging to the TD-IZQ model.

For the TD-IZQ model, the results of the inspection of 58 modules are displayed in detail in Table A1. The MVS correctly adjusted 83% of the analysed modules. The rest did not meet the acceptance range for any of the tooling positions. The cut-off sharpness feature was correct for 97% of the modules and the colour features were within the range for the 83% percentage of red and the 95% for the percentage of blue. Furthermore, most of the modules reached their optimal sharpness and colour features in positions 4 and 5. Figure 12 shows these results.

**Figure 13:**
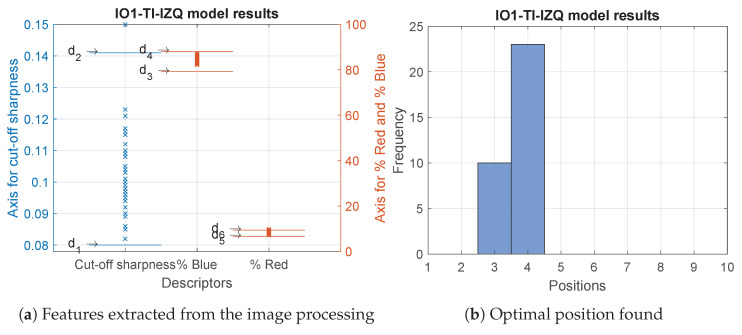
Results of the analysed modules belonging to the IO1-TI-IZQ model.

For the IO1-TI-IZQ model, the results were quite similar. In this case, of the 37 modules, 89% were optimally adjusted. Almost for all the modules the three features were within the established acceptance ranges. The optimal position that was repeated most frequently was number 4. These results can be seen in Figure 13 and in detail in Table A2.

**Figure 14:**
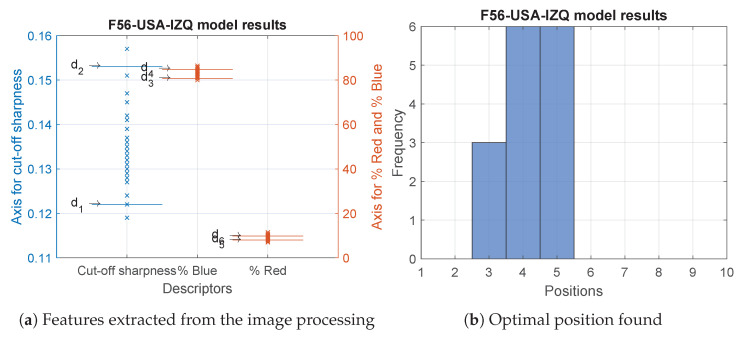
Results of the analysed modules belonging to the F56-USA-IZQ model.

The worst results were obtained with the F56-USA-IZQ model. Of a total of 39 modules, 38% had the three features within their acceptance ranges, while 92% showed an acceptable cut-off sharpness; for a little more than half of them, 54%, it was not possible to be within the range of the percentage of red. This was the main reason why the MVS was not able to adjust 62% of the modules. The results for the percentage of blue were better as 72% of the modules were within the range. Once the optimal position was adjusted, the most repeated were number 4 and number 5. Figure 14 and Table A3 show the former data.

It is worth mentioning that all the automatically adjusted BiLED modules followed the ECE R112 regulation when checked in a photometric bench.

## 5. Conclusions

The purpose of this work is to present a machine vision system for the automatic adjustment of LED modules for automotive lighting. One of the advantages is the flexibility to adapt to new models. This is possible thanks to the development of a component-based software architecture together with an adaptive hardware design and an iterative optimal adjustment methodology based on learned positions.

To check the functionality of the system, it was calibrated with three models of LED modules with an adjustment according to regulation and customer criteria. In total, the cut-off line of more than 100 modules was analysed by the MVS to generate the knowledge base. Once the machine had been calibrated, it was used to automatically adjust newly manufactured modules of these three models. The optimal position was computed via a positioning algorithm which used, as a decision base, the features extracted from the cut-off line of the modules. The image processing features were the cut-off sharpness and colour measurements in the CIE diagram from the points placed on the border of the cut-off line.

With these data, most of the modules were optimally adjusted for two of the three models of BiLED modules. The remainder module had the worst results because of the narrow range defined for one of the colour measurements. After the automatic assembly, all of the adjusted modules were analysed in a photometric bench to assure that 100% followed the ECE R112 regulation.

## Figures and Tables

**Table 1:** Features obtained for each unit inspected during the calibration process.

	TD-IZQ Model			IO1-TI-IZQ Model			F56-USA-IZQ		
**n**	${CW}_{n}^{std}$	${CL}_{1}^{std}$	${CL}_{2}^{std}$	${CW}_{n}^{std}$	${CL}_{1}^{std}$	${CL}_{2}^{std}$	${CW}_{n}^{std}$	${CL}_{1}^{std}$	${CL}_{2}^{std}$
1	0.106	7.710	83.755	0.112	8.175	85.188	0.122	8.300	81.830
2	0.101	8.911	84.428	0.094	9.140	86.252	0.136	8.790	83.670
3	0.099	9.288	86.450	0.096	7.543	80.516	0.139	8.810	82.680
4	0.093	7.671	83.491	0.121	7.798	80.488	0.13	8.950	82.660
5	0.095	8.321	83.993	0.102	8.246	85.275	0.141	8.610	82.150
6	0.104	8.367	85.995	0.081	8.215	85.877	0.148	8.520	80.680
7	0.106	8.735	86.558	0.141	7.091	79.242	0.132	9.360	83.160
8	0.104	8.394	86.281	0.107	7.379	79.612	0.137	9.070	83.690
9	0.089	13.268	84.475	0.110	7.543	81.971	0.130	9.820	84.820
10	0.093	8.186	82.262	0.091	7.743	82.171	0.140	9.160	82.790
11	0.073	21.520	78.560	0.141	9.348	82.426	0.131	8.40	83.080
12	0.090	11.923	85.276	0.080	9.048	88.057	0.144	9.570	84.450
13	0.115	9.573	86.048	0.088	8.673	87.041	0.131	9.140	83.220
14	0.087	19.095	80.774	0.091	6.898	82.281	0.138	8.770	82.650
15	0.096	8.637	84.160	0.092	7.355	81.132	0.136	8.570	82.070
16	0.103	8.101	83.449	0.118	6.740	82.017	0.135	8.830	83.130
17	0.090	10.141	87.980	0.117	7.410	81.918	0.136	9.790	83.760
18	0.093	7.918	83.813	0.097	8.859	86.529	0.142	8.600	81.880
19	0.096	9.821	85.977	0.104	7.749	82.989	0.142	8.060	81.310
20	0.089	8.980	84.380	0.113	7.598	81.669	0.135	8.730	83.080
21	0.117	8.727	79.788	0.099	8.350	85.437	0.13	8.000	82.740
22	0.119	7.332	82.822	0.083	7.797	81.96	0.136	9.470	83.030
23	0.091	13.343	84.209	0.099	8.609	86.016	0.136	8.620	82.710
24	0.089	18.684	80.522	0.091	6.876	81.534	0.153	9.140	82.640
25	0.103	14.583	83.358	0.116	7.302	81.947			
26	0.100	8.181	85.592	0.105	8.383	86.926			
27	0.112	10.786	86.113	0.093	8.247	84.69			
28	0.115	8.059	85.042	0.117	6.792	81.028			
29	0.092	12.739	83.006	0.085	8.799	86.838			
30	0.088	13.322	83.622	0.111	6.761	82.624			
31	0.078	25.687	70.258	0.097	7.723	82.142			
32	0.116	8.440	85.882	0.099	7.171	81.134			
33	0.106	15.711	82.083	0.103	8.274	85.487			
34	0.090	14.901	82.231	0.106	7.291	82.135			
35	0.101	9.203	83.271	0.108	8.425	85.643			
36	0.091	13.371	84.116	0.125	7.253	81.102			
37	0.090	12.300	83.759	0.098	8.039	85.682			
38	0.068	19.253	79.976	0.097	7.012	81.718			
39	0.093	13.674	83.290	0.089	8.771	85.739			
40	0.110	9.316	85.419	0.102	6.802	80.711			
41				0.106	7.697	85.693			
42				0.104	6.963	81.239			
43				0.117	6.572	81.038			
44				0.114	6.925	81.497			
45				0.107	6.601	81.304			
46				0.117	7.239	80.902			
47				0.097	7.675	85.742			
48				0.098	8.753	85.947			

**Table 2:** Acceptance ranges obtained for each model.

Model	Cut-Off Sharpness ($d_{1}-d_{2}$)	Percentage of Red ($d_{3}-d_{4}$)	Percentage of Blue ($d_{5}-d_{6}$)
TD-IZQ	(0.068–0.119)	(7.330–25.680)	(70.250–87.980)
IO1-TI-IZQ	(0.080–0.141)	(6.570–9.340)	(79.240–88.050)
F56-USA-IZQ	(0.122–0.153)	(8.000–9.820)	(80.680–84.820)

## Data Availability

The data presented in this study are available on request from the corresponding author. The data are not publicly available due to confidentiality reasons derived from the project members.

## Abbreviations

The following abbreviations are used in this manuscript:

HB	High-beam
ECE	Economic Commission for Europe
GUI	Graphical User Interface
LED	Light Emitting Diode
LB	Low-beam
LHD	Left Hand Traffic
MVS	Machine Vision System
PLC	Programmable Logic Controller
RHD	Right Hand Traffic
ROI	Region Of Interest
UML	Unified Modelling Language
WLLED	White Light LED

## Appendix A

### Appendix A.1

**Table A1:** Results of the analysed modules belonging to the TD-IZQ model.

Module	Cut-Off Sharpness	% Blue	% Red	Optimal Position
1	0.130	79.270	5.830	-
2	0.090	78.110	18.500	4
3	0.092	81.100	15.350	4
4	0.110	82.180	6.430	-
5	0.120	80.340	5.410	-
6	0.100	80.940	6.530	-
7	0.100	79.870	6.060	-
8	0.110	82.940	6.270	-
9	0.097	84.030	12.560	4
10	0.100	82.880	13.850	4
11	0.110	84.130	13.860	4
12	0.097	76.510	21.460	4
13	0.103	80.890	15.840	4
14	0.107	82.760	15.560	4
15	0.090	82.750	14.470	4
16	0.086	75.450	23.250	5
17	0.079	73.280	25.670	5
18	0.085	82.310	16.080	5
19	0.092	76.670	22.040	5
20	0.093	80.920	17.610	5
21	0.094	67.570	26.420	-
22	0.096	84.110	12.910	4
23	0.089	81.930	15.580	5
24	0.099	80.720	17.490	5
25	0.086	74.300	23.410	6
26	0.099	82.920	14.980	4
27	0.110	79.850	17.490	5
28	0.083	80.420	18.820	5
29	0.093	73.240	24.760	5
30	0.097	80.290	17.530	5
31	0.101	84.060	12.970	4
32	0.084	79.080	19.740	5
33	0.083	74.250	25.250	5
34	0.080	81.460	14.020	5
35	0.112	83.930	12.870	4
36	0.103	81.250	17.130	4
37	0.086	75.740	20.800	5
38	0.100	68.550	28.350	-
39	0.091	78.320	17.330	5
40	0.076	76.280	21.030	5
41	0.099	81.200	17.430	6
42	0.076	80.840	13.410	5
43	0.078	72.060	23.380	5
44	0.087	74.680	24.220	5
45	0.079	66.120	25.870	-
46	0.083	74.600	21.830	6
47	0.089	71.520	25.920	-
48	0.080	79.290	16.360	6
49	0.089	79.650	19.140	5
50	0.078	74.450	22.460	5
51	0.078	78.340	19.200	5
52	0.078	79.350	18.850	5
53	0.089	75.200	22.500	5
54	0.077	73.920	24.350	5
55	0.083	76.400	21.030	5
56	0.085	75.390	24.840	7
57	0.081	72.430	22.710	5
58	0.082	79.670	19.420	5

### Appendix A.2

**Table A2:** Results of the analysed modules belonging to the IO1-TI-IZQ model.

Module	Cut-Off Sharpness	% Blue	% Red	Optimal Position
1	0.092	83.060	8.310	3
2	0.098	84.290	7.560	4
3	0.096	85.390	9.420	-
4	0.095	82.680	8.620	4
5	0.110	84.060	7.490	3
6	0.123	82.790	7.380	3
7	0.115	87.100	9.150	4
8	0.090	84.310	7.780	4
9	0.097	87.280	8.360	4
10	0.092	86.100	9.020	4
11	0.105	85.490	8.990	4
12	0.082	86.610	8.050	4
13	0.105	82.250	7.570	4
14	0.094	86.300	8.250	4
15	0.101	86.060	8.050	4
16	0.150	84.020	8.510	-
17	0.099	84.220	8.380	4
18	0.078	86.190	9.030	-
19	0.101	83.800	7.630	4
20	0.086	86.210	7.890	4
21	0.085	85.520	8.390	4
22	0.092	86.130	9.880	-
23	0.109	85.070	7.860	3
24	0.103	84.280	7.990	3
25	0.100	84.760	7.560	4
26	0.089	83.620	7.190	3
27	0.108	83.660	7.610	4
28	0.099	82.170	7.170	4
29	0.098	84.000	8.260	3
30	0.117	83.800	8.970	4
31	0.104	83.310	8.250	4
32	0.121	82.980	7.640	4
33	0.116	82.890	8.100	4
34	0.097	85.400	8.230	3
35	0.112	83.740	8.210	4
36	0.105	84.740	7.540	3
37	0.108	84.960	8.310	3

### Appendix A.3

**Table A3:** Results of the analysed modules belonging to the F56-USA-IZQ model.

Module	Cut-Off Sharpness	% Blue	% Red	Optimal Position
1	0.131	82.720	7.450	-
2	0.130	80.730	6.930	-
3	0.130	81.820	7.420	-
4	0.119	84.450	10.740	-
5	0.128	81.020	6.930	-
6	0.127	81.890	7.400	-
7	0.127	84.290	8.260	3
8	0.134	83.640	9.500	4
9	0.151	83.520	11.500	-
10	0.132	84.920	10.040	-
11	0.135	83.030	9.000	3
12	0.136	81.470	8.570	3
13	0.128	84.530	9.550	4
14	0.127	85.010	9.400	-
15	0.130	82.980	7.950	-
16	0.136	84.160	11.380	-
17	0.131	84.000	9.060	5
18	0.124	85.530	9.740	-
19	0.137	84.960	9.520	-
20	0.141	83.800	9.890	-
21	0.133	84.430	9.750	5
22	0.133	85.600	10.130	-
23	0.127	85.600	9.670	-
24	0.145	81.810	8.890	4
25	0.142	82.650	9.300	5
26	0.141	85.590	10.140	-
27	0.151	81.480	9.120	4
28	0.131	85.880	10.790	-
29	0.147	81.230	8.640	4
30	0.141	84.090	9.190	5
31	0.132	86.430	10.070	-
32	0.129	84.420	9.830	-
33	0.130	82.270	9.010	5
34	0.157	82.940	9.460	-
35	0.139	84.950	10.280	-
36	0.142	83.630	9.430	5
37	0.135	83.590	10.140	-
38	0.142	81.540	8.140	4
39	0.122	79.970	9.410	-
